# Digestive tract toxicity associated with exposure to 2,4-dichlorophenoxyacetic acid in rats

**DOI:** 10.1590/1414-431X2022e12350

**Published:** 2022-09-12

**Authors:** V.C.B.S. Mariotti, I.Z.F. Naufal, I.A.R. Amorim, J.L.S. Parizi, G.A. Nai

**Affiliations:** 1 Programa de Pós-Graduação em Ciências da Saúde, Universidade do Oeste Paulista, Presidente Prudente, SP, Brasil; 2 Departamento de Medicina, Universidade do Oeste Paulista, Presidente Prudente, SP, Brasil; 3 Faculdade de Medicina, Universidade do Oeste Paulista, Presidente Prudente, SP, Brasil; 4 Departamento de Patologia, Universidade do Oeste Paulista, Presidente Prudente, SP, Brasil

**Keywords:** Gastrointestinal tract, Esophageal diseases, Environmental health surveillance, Chronic toxicity, Exposure to pesticides

## Abstract

2,4-Dichlorophenoxyacetic acid (2,4-D) is a herbicide of the chlorophenoxy class and the second most widely used herbicide applied to several different crops worldwide. Environmental factors, especially those related to diet, strongly affect the risk of developing cancer of the gastrointestinal tract. There is currently no evidence to determine whether there is an association between 2,4-D exposure and gastrointestinal disorders. We evaluated the histological effect of chronic oral and inhalation exposure to 2,4-D on the digestive tract of rats. Eighty male adult albino Wistar rats were divided into 8 groups (n=10): two control groups, one for inhalation and one for oral exposure, and 6 groups exposed orally or by inhalation at three different concentrations of 2,4-D [3.71×10^-3^ grams of active ingredient per hectare (gai/ha), 6.19×10^-3^ gai/ha, and 9.28×10^-3^ gai/ha]. The animals were exposed for 6 months. The esophagus, stomach, and intestine were collected for histopathological analysis. Animals exposed to 2,4-D had hyperkeratosis of the esophagus, regardless of the exposure route. All animals exposed to a higher concentration of 2,4-D orally presented mild dysplasia of the large intestine. In the small intestine, most animals exposed to moderate and high concentrations of 2,4-D had mild dysplasia. No gastric changes were observed in any of the groups studied. Chronic exposure to 2,4-D, especially at moderate and high concentrations, regardless of the exposure route, caused reactive damage to the esophagus (hyperkeratosis) and dysplastic changes to the intestine.

## Introduction

Pesticides for pest and weed control have been used for many decades to assist the growth and development of crops and pastures (1). The widespread use of pesticides in agriculture, whether to eliminate pests or regulate crop growth, has important toxicological effects. Poisoning of animals or humans with such agents can occur after exposure to soil, air, water, or failure to follow safety instructions (1).

2,4-Dichlorophenoxyacetic acid (2,4-D) is a synthetic auxin of the class of chlorophenoxy acidic herbicides and was the first selective herbicide developed to control dicot plants and broadleaf plants, cultivation leaves, and weeds (2). 2,4-D has been commercialized since 1944 and is one of the most widely used herbicides in the world due to its general applicability and low cost. There are currently more than 600 products containing 2,4-D on the market. This herbicide is sold in various formulations under a wide variety of brands and is found, for example, in commercial mixes of lawn herbicides (3). In addition, 2,4-D has a long residual activity time in soils and waters, persisting for several months due to its low microbiological activity (4), leading to contamination of humans and animals over time.

During the Vietnam War, in the 1950s and 1970s, 2,4-D was used by the US Air Force as a defoliating agent, along with 2,4,5-trichlorophenoxyacetic acid (2,4,5-T) and pentachlorophenol (PCF), forming the “agent orange” (5). Since the mid-1990s, Korean researchers have been investigating health issues related to “agent orange”. As decades have passed since Korean military units withdrew from Vietnam, the late investigation of this herbicide presented some challenges. However, some studies revealed that high exposure to “agent orange” was associated with a significantly higher prevalence of cancers (colon cancer, leukemia, and multiple myeloma), circulatory diseases (hypertension, cerebral infarction, and peripheral vasculopathy), neuromuscular diseases (peripheral neuropathy, multiple nerve palsy, and multiple sclerosis), skin diseases, and dyslipidemia (5).

A cohort study by the International Agency for Research on Cancer (IARC) of production workers and sprayers of phenoxy herbicide and chlorophenol found less digestive system disease and cirrhosis mortality in exposed workers than in unexposed controls. Mortality results up to 2001 for the Seveso cohort in Italy did not show an increase in deaths related to digestive diseases or specifically to cirrhosis in those exposed to 2,4-D. Several mortality studies from various occupational cohorts of people exposed to 2,4-D have been inconsistent but they generally found no significant increase in deaths from ulcers or cirrhosis (6). Interpretation of individual studies is difficult due to the lack of information about alcohol consumption and other risk factors. In the studies that showed the strongest association between exposure to 2,4-D and gastrointestinal disease (specifically cirrhosis), exposure to alcohol was not excluded (6).

In view of the inconsistencies in determining whether there is an association between 2,4-D exposure and gastrointestinal diseases and the importance of environmental factors related to diet as a risk for developing gastrointestinal cancer, the aim of this study was to evaluate the possible damage to the digestive tract during oral and inhalation chronic exposure to the herbicide 2,4-D in rats, simulating environmental exposure (occupational and non-occupational) to similar concentrations of human exposure.

## Material and Methods

### Ethical approval

This study was approved by the Animal Use Ethics Committee of the Universidade do Oeste Paulista (UNOESTE; Protocol No. 6032). All applicable international, national, and institutional guidelines for the care and use of animals were followed.

### Test chemical

Exposure was conducted with 2,4-D (Nortox SA, Brazil), with the following composition described in the product insert: dimethylamine salt of (2,4-dichlorophenoxy) acetic acid (2,4-D): 806 g/liter (80.6% m/v), 2.4 D acid equivalent: 670 g/liter (67.0% m/v), and inert ingredients: 424 g/liter (42.4% m/v).

### Animal protocol

To carry out the experiments, 80 adult (initial age of 90 days) male Wistar rats weighing 200-250 g were supplied by the Central Vivarium of UNOESTE and allocated to large plastic cages (two animals per cage) at an average temperature of 22±2°C with 12-h light cycles from 7:00 am to 7:00 pm (light period) and 7:00 pm to 7:00 am (dark period).

The animals were randomly divided into eight groups (n=10/group): I: Inhalation control group: exposed to nebulization with distilled water; O: Oral control group: received nebulized feed with distilled water; 2,4-D LI: Group exposed to a low concentration of 2,4-D by inhalation; 2,4-D LO: Group received nebulized feed with a low concentration of 2,4-D; 2,4-D MI: Group exposed to a moderate concentration of 2,4-D by inhalation; 2,4-D MO: Group received nebulized feed with a moderate concentration of 2,4-D; 2,4-D HI: Group exposed to a high concentration of 2,4-D by inhalation; 2,4-D HO: Group received nebulized feed with a high concentration of 2,4-D.

### Exposure to 2,4-D protocol

For this study, we selected the lowest, average, and highest used concentrations of 2,4-D in crops, according to the product insert, which were adjusted to the size of the nebulization box: 3.71×10^-3^ g of active ingredient per hectare (gai/ha) of 2,4-D (corresponding to 20.69 ppm), considered a low concentration; 6.19×10^-3^ gai/ha of 2,4-D (corresponding to 34.63 ppm), considered a moderate concentration; and 9.28×10^-3^ gai/ha of 2,4-D (corresponding to 51.66 ppm), considered a high concentration.

For nebulization, two boxes (32×24×32 cm) were used, one for the control group and another for the groups exposed to 2,4-D. Each box was connected to an ultrasonic nebulizer (Pulmosonic Star^®^, Soniclear Ind. Com. Imp. Exp. Ltda., Brazil). The animals and feed were exposed until the entire solution was nebulized (approximately 15 min) (7).

**Figure 1:**
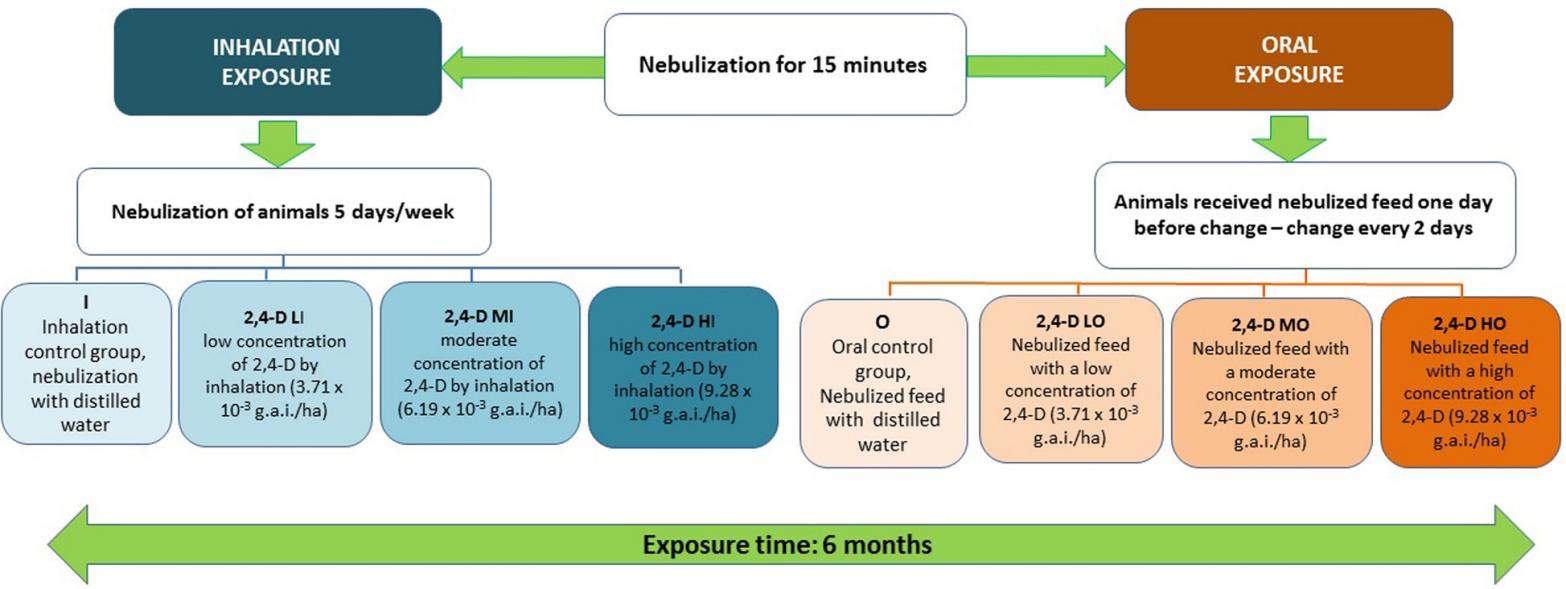
Experimental study design. g.a.i./ha: grams of active ingredient per hectare.

The animals exposed by inhalation were nebulized daily for five consecutive days during the week. The feed of the animals exposed orally was nebulized one day before being offered and changed every two days (8) (Figure 1). The residual feed was weighed at each change to control intake by the animals. The animals were weighed monthly until the end of the experiment.

Animals in all groups were exposed for 6 months. Euthanasia was performed at the end of the experiment with intraperitoneal injection of sodium thiopental (Syntec, Brazil) at a dose of 100 mg/kg of weight.

### Histopathological analysis

After euthanasia, the esophagus, stomach, and small and large intestines of each animal were removed. Three cross-sections of the esophagus (proximal, middle, and distal third), one from the gastric fundus, body, and antrum, one cross-section of the jejunum, one cross-section of the ileum, and two random cross-sections of the large intestine were fixed in 10% formaldehyde (Cinética Indústria Química, Brazil) for 24 h and subjected to standard histological processing, including paraffin embedding (Dynamic Analytical Reagents, Brazil). Five micrometer sections of each organ were obtained by a LEICA RM2265 microtome (Leica Biosystems Nussoch GmbH, Germany) and stained using the hematoxylin-eosin (HE) method (Dolles, Brazil).

The histopathological analysis of the groups was conducted by a pathologist blinded to the treatment using a standard optical microscope (NIKON Labophot, Japan). Three serial cuts from each organ were analyzed. The general parameters evaluated in the esophagus, stomach, and small and large intestines with their respective scores were interstitial inflammatory infiltrate (0=absent, 1=mild, 2=moderate, 3=intense) and type of inflammatory cell present (polymorphonuclear and/or mononuclear); tissue congestion (0=absent, 1=mild, 2=moderate, 3=severe); tissue necrosis (0=absent; 1=present and focal, 2=present and diffuse); vascular necrosis (0=absent, 1=present); non-neoplastic changes in the mucosa (atrophy, hyperplasia; 0=absent, 1=present); dysplastic lesions (0=absent, 1=mild dysplasia, 2=moderate dysplasia, 3=severe dysplasia), and presence of benign and malignant neoplastic lesions (0=absent, 1=benign, 2=malignant) (9).

To assess epithelial dysplasia, the following parameters were evaluated: epithelial maturation, gland architecture, and cytological criteria (vesicular nuclei with irregular chromatin, voluminous, hyperchromatic nuclei, increased nucleus/cytoplasm ratio, loss of nuclei polarity, presence of prominent nucleoli, atypical mitoses) (10).

The following parameters were also specifically analyzed (9): esophagus: hyperkeratosis (defined as an increase in the stratum corneum) (0=absent, 1=mild, 2=moderate, 3=severe), parakeratosis (defined as abnormal maturation of the squamous epithelium with the presence of nucleated cells in the stratum corneum) (0=absent, 1=focal, 2=diffuse), metaplasia (0=absent, 1=focal, 2=diffuse); intestine: lymphoid hyperplasia (0=absent, 1=present).

### Morphometric analysis of the esophagus

The following histomorphometric analyses were performed on the esophagus: measurement of the total thickness of the esophageal epithelium - measurements were performed on two areas of the whole epithelium per animal using ImageJ software (National Institutes of Health (NIH), USA, available at http:/rsbweb.nih.gov/ij/) (9); measurement of the thickness of the stratum corneum - measurements were performed on two areas of the stratum corneum per animal using ImageJ software (9); NORs (nucleoli-organizing regions) [which is a marker of cell proliferation (11)] were counted - deparaffinized cuts were stained with silver impregnation and counterstained with Van Gieson (“light green”) (Merck, Germany) according to the technique of Ploton et al. (12), and 100 nuclei were evaluated per animal.

### Statistical analysis

Qualitative variables were analyzed using the likelihood ratio. The variables weight and feed intake showed homogeneity of variances (Levene's test, P>0.05) and groups were compared with analysis of variance. The epithelium thickness variable showed a normal distribution (Kolmogorov-Smirnov test, P>0.05) and homogeneity of variances (Levene test, P>0.05) and groups were compared with analysis of variance, followed by the minimum difference test for multiple comparisons. The data for number of NORs did not show normality (Kolmogorov-Smirnov test, P<0.05) or homogeneity of variances (Levene test, P<0.05), and so the Kruskal-Wallis test was used to compare the distribution points, followed by the Dunn test for multiple comparisons. These variables were correlated with Spearman's nonparametric correlation. To verify the agreement between dysplasia in the large and small intestines, the kappa agreement coefficient was used. The effect size was also evaluated by Cohen's d, where values <0.19 are considered insignificant, 0.20-0.49 are considered small, 0.50-0.79 medium, 0.80-1.29 large, and >1.30 very large (13).

The data were processed with software SPSS 23.0 (IBM, USA). All statistical tests were considered significant at the 5% level (P<0.05).

## Results

### Mortality

One animal in the 2,4-D HO group died during the study due to an ear canal infection.

### Animal weight and feed consumption

There was no difference in the animals' weight and feed consumption during the experiment among the study groups (P>0.05).

### Esophagus

**Figure 2:**
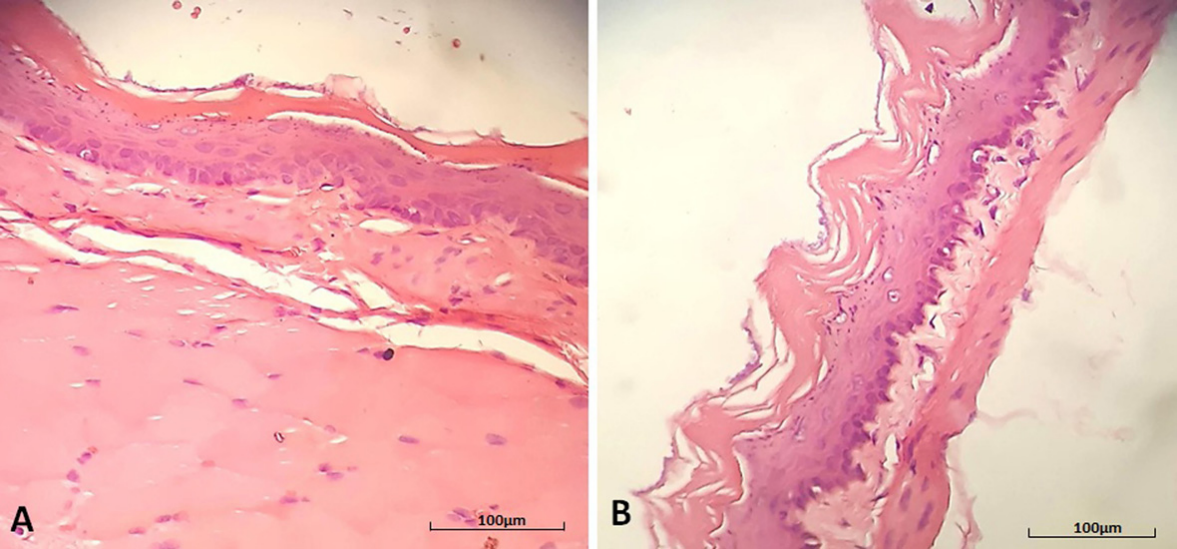
Photomicrographs of the esophagus. **A**, Normal epithelium (animal of the inhalation control group). **B**, Epithelium with increased thickness of the corneal layer (hyperkeratosis) from an animal of the group exposed to a low concentration of 2,4-dichlorophenoxyacetic acid by inhalation. Hematoxylin-eosin staining; scale bar 100 μm.

**Figure 3:**
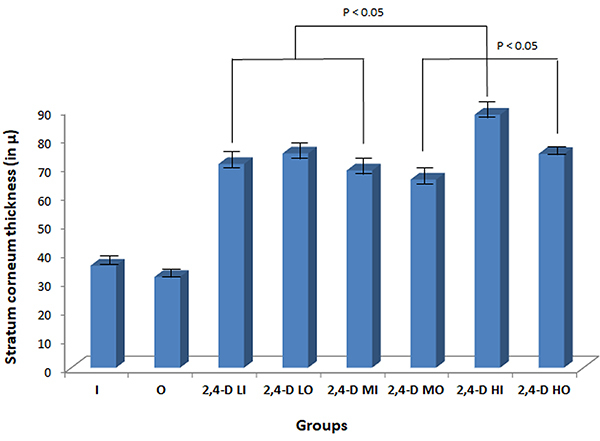
Thickness of the stratum corneum (in µ) in the experimental groups (n=79). Data are reported as mean±SD (ANOVA). I: Inhalation control group; O: Oral control group; 2,4-D LI: Group exposed to low concentration of 2,4-dichlorophenoxyacetic acid (2,4-D) by inhalation; 2,4-D LO: Group exposed to low concentration of 2,4-D orally; 2,4-D MI: Group exposed to moderate concentration of 2,4-D by inhalation; 2,4-D MO: Group exposed to moderate concentration of 2,4-D orally; 2,4-D HI: Group exposed to high concentration of 2,4-D by inhalation; 2,4-D HO: Group exposed to a high concentration of 2,4-D orally.

Hyperkeratosis was observed in most animals exposed to 2,4-D and was independent of herbicide concentration and exposure route (oral or inhaled) (P<0.05) (Table 1, Figure 2). The mean stratum corneum thickness of the esophagus in the unexposed groups was 33.70 (standard deviation (SD): ±2.77) μm, and in the groups exposed to 2,4-D it was 73.92 (±7.87) μm. There was a statistically significant difference in mean thickness of the stratum corneum of the 2,4-D LI × 2,4-D HI and 2,4-D MI × 2,4-D HI groups and of the 2,4-D MO × 2,4-D HO groups (P<0.05). The animals in groups 2,4-D HI showed the greatest thickness measurement (Figure 3). With a Cohen’s d of 6.817449, a very large difference in stratum corneum thickness was found between the groups exposed and not exposed to 2,4-D.

**Table 1:** Incidence of hyperkeratosis in the esophageal epithelium in the experimental groups (n=79).

Groups	Hyperkeratosis	Groups	Hyperkeratosis
Inhalation		Oral	
Control	0/10 (0%)^Aa^	Control	0/10 (0%)^Aa^
2,4-D LI	8/10 (80%)^Ab^	2,4-D LO	8/10 (80%)^Ab^
2,4-D MI	7/10 (70%)^Ab^	2,4-D MO	10/10 (100%)^Ab^
2,4-D HI	10/10 (100%)^Ab^	2,4-D HO	9/9 (100%)^Ab^

Data are reported as number and percent. 2,4-D LI: Group exposed to low concentration of 2,4-dichlorophenoxyacetic acid (2,4-D) by inhalation; 2,4-D LO: Group exposed to low concentration of 2,4-D orally; 2,4-D MI: Group exposed to the moderate concentration of 2,4-D by inhalation; 2,4-D MO: Group exposed to the moderate concentration of 2,4-D orally; 2,4-D HI: Group exposed to high concentration of 2,4-D by inhalation; 2,4-D HO: Group exposed to a high concentration of 2,4-D orally. Upper-case letters compare the inhaled and oral groups within the same concentration. Lower-case letters compare different concentrations within the same exposure route. Different letters indicate P<0.05 (Kruskal-Wallis test).

No congestion, inflammation, tissue necrosis, vascular necrosis, atrophy, hyperplasia, metaplasia, parakeratosis, or dysplastic or neoplastic lesions were observed in any of the animals evaluated.

### Total thickness of the esophageal epithelium

**Figure 4:**
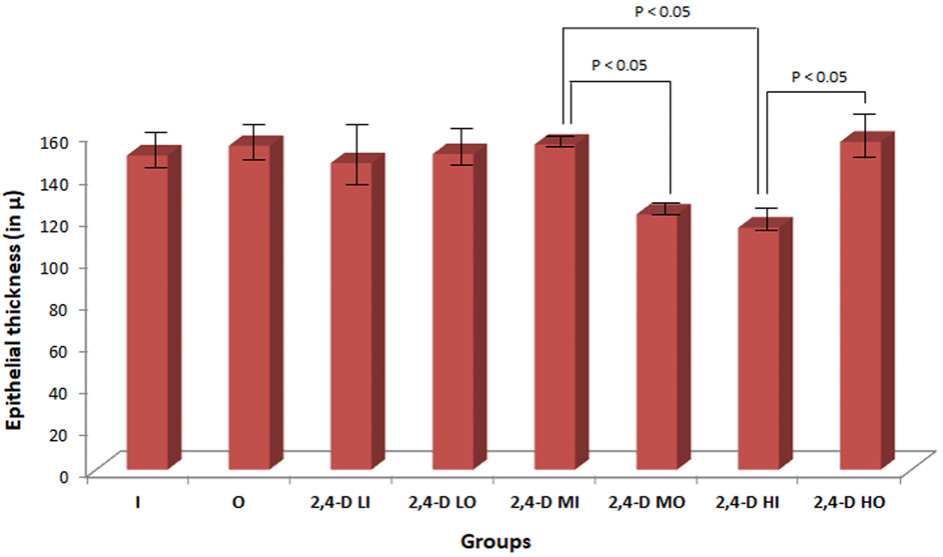
Thickness of the esophagus epithelium (in µ) in the experimental groups (n=79). Data are reported as means±SD (ANOVA). I: Inhalation control group; O: Oral control group; 2,4-D LI: Group exposed to low concentration of 2,4-dichlorophenoxyacetic acid (2,4-D) by inhalation; 2,4-D LO: Group exposed to low concentration of 2,4-D orally; 2,4-D MI: Group exposed to moderate concentration of 2,4-D by inhalation; 2,4-D MO: Group exposed to moderate concentration of 2,4-D orally; 2,4-D HI: Group exposed to high concentration of 2,4-D by inhalation; 2,4-D HO: Group exposed to a high concentration of 2,4-D orally.

The mean total epithelial thickness of the esophagus in the unexposed groups was 152.24±3.28 μm and in the exposed groups was 140.96±17.75 μm. There was a statistically significant difference in means of the 2,4-D MI × 2,4-D HI and 2,4-D MI × 2,4-D MO groups and of the 2,4-D HI × 2,4-D HO groups (P<0.05). The animals in groups 2,4-D MO and 2,4-D HI showed the lowest measures (Figure 4). With a Cohen’s d of 0.883761, the exposed animals showed a large difference in total epithelial thickness with non-exposed groups.

### Number of NORs in the esophagus

**Figure 5:**
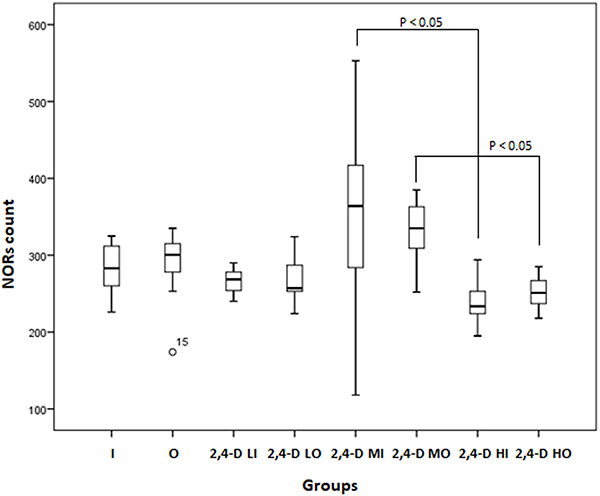
Number of nucleoli-organizing regions (NORs) in the esophageal epithelium in the experimental groups (n=79). Data are reported as median and interquartile ranges (Kruskal-Wallis test). Groups: I: Inhalation control group; O: Oral control group; 2,4-D LI: Group exposed to low concentration of 2,4-dichlorophenoxyacetic acid (2,4-D) by inhalation; 2,4-D LO: Group exposed to low concentration of 2,4-D orally; 2,4-D MI: Group exposed to moderate concentration of 2,4-D by inhalation; 2,4-D MO: Group exposed to moderate concentration of 2,4-D orally; 2,4-D HI: Group exposed to high concentration of 2,4-D by inhalation; 2,4-D HO: Group exposed to a high concentration of 2,4-D orally.

There was a statistically significant difference between the NORs in the 2,4-D MI × 2,4-D HI groups and between 2,4-D MO × 2,4-D HO, where groups exposed to a moderate concentration had more NORs than groups exposed to a high concentration of 2,4-D (P<0.05) (Figure 5).

The mean number of NORs in the esophageal epithelium of the unexposed groups was 285.68±39.29, and in the exposed groups it was 282.97±67.38. With a Cohen's d of 0.049136, the exposed animals showed a non-significant difference in NORs count from those not exposed to 2,4-D.

### Correlation between epithelial thickness and NORs in the esophagus

**Figure 6:**
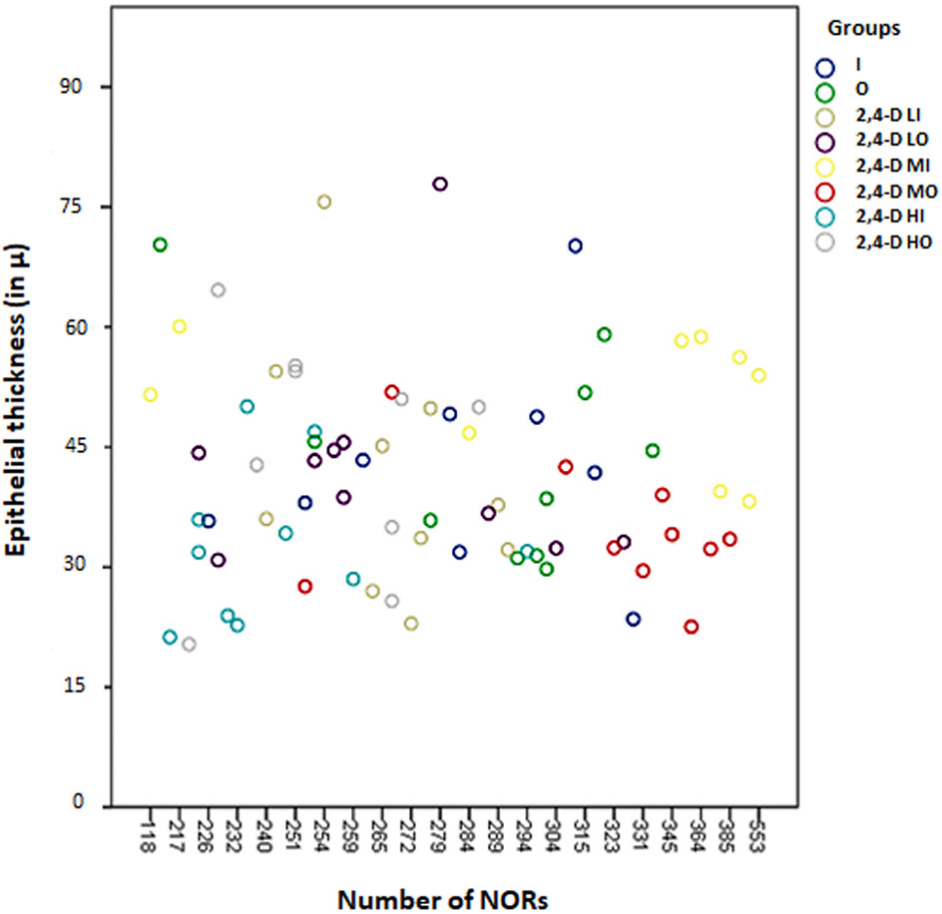
Correlation between total epithelial thickness (in µ) and the number of nucleoli-organizing regions (NORs) (per mm^2^) in the esophageal epithelium in the experimental groups (ρ =-0.010; P=0.934) (Spearman's nonparametric correlation). Groups: I: Inhalation control group; O: Oral control group; 2,4-D LI: Group exposed to low concentration of 2,4-dichlorophenoxyacetic acid (2,4-D) by inhalation; 2,4-D LO: Group exposed to low concentration of 2,4-D orally; 2,4-D MI: Group exposed to moderate concentration of 2,4-D by inhalation; 2,4-D MO: Group exposed to moderate concentration of 2,4-D orally; 2,4-D HI: Group exposed to high concentration of 2,4-D by inhalation; 2,4-D HO: Group exposed to a high concentration of 2,4-D orally.

There was no correlation between epithelial thickness and number of NORs in the esophagus (ρ=-0.010; P=0.934) (Figure 6).

### Stomach

No gastric changes were observed in any of the animals evaluated.

### Intestine

**Figure 7:**
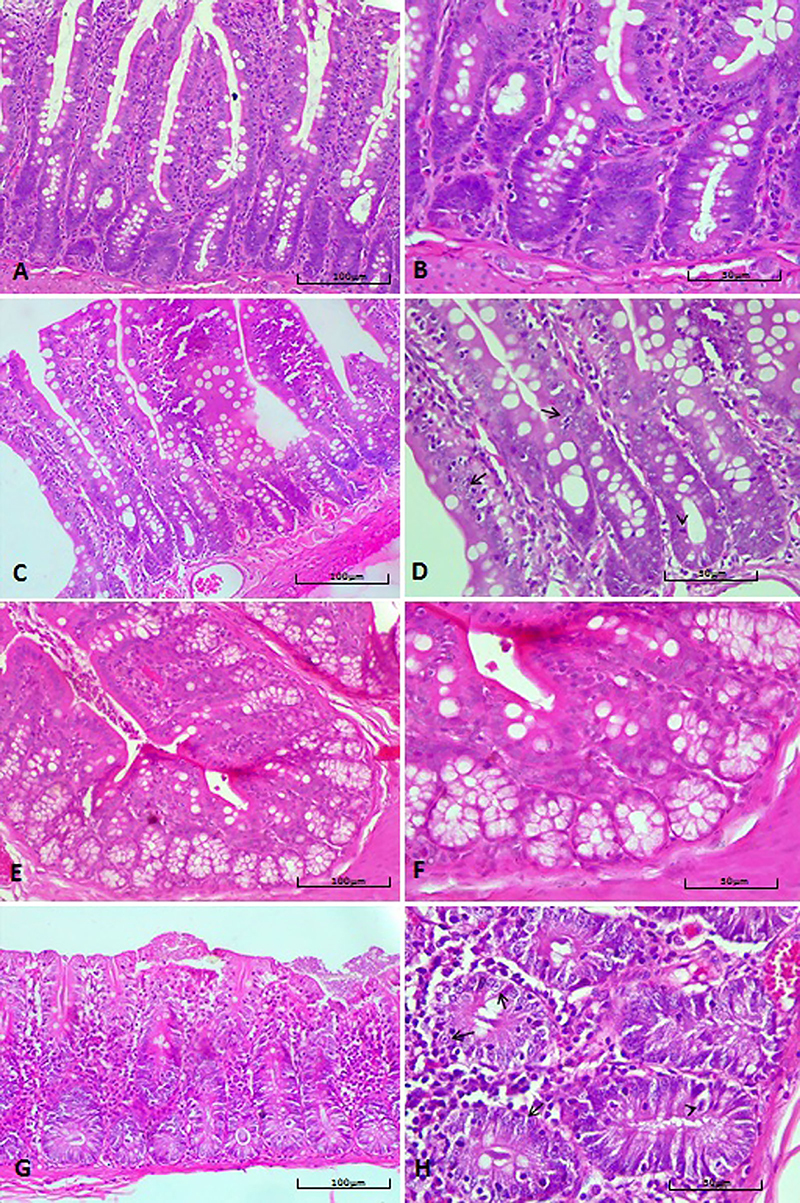
**A**-**D**, Microphotographs of the small intestine: **A**, Normal mucosa (animal from the inhalation control group). **B**, Detail of the mucosa. **C**, Mucosa with mild dysplasia. **D**, Loss of polarity of nuclei, which have a vesicular form with evident nucleoli (arrows) and atypical mitotic figure (arrowhead) (animal of the group exposed to moderate concentration of 2,4-dichlorophenoxyacetic acid (2,4-D) by inhalation). **E**-**H**, Microphotographs of the large intestine: **E**, Normal mucosa (animal of the inhalation control group). **F**, Detail of the mucosa of the large intestine. **G**, Mucosa with mild dysplasia. **H**, Absence of goblet cells, loss of polarity of nuclei, which are vesicular with irregular chromatin and evident nucleoli (arrows) and atypical mitosis (arrowhead) (animal from the group exposed to moderate concentration of 2,4-D orally). Hematoxylin-eosin staining; scale bars 100 and 50 μm.

Animals exposed to moderate and high concentrations of 2,4-D had mild dysplasia in both the small and large intestines (P<0.05), with a higher incidence in animals exposed orally to high concentrations (P<0.05) (Table 2, Figure 7). There was a higher incidence of mild dysplasia in the small intestine than in the large intestine among animals exposed to moderate concentrations (oral and inhalation), but the kappa coefficient showed that these proportions were the same in the small and large intestines. No moderate or severe dysplasia was observed in any of the animals evaluated.

**Table 2:** Incidence of mild dysplasia in the small and large intestines in the experimental groups (n=79).

Groups	Mild dysplasia	Groups	Mild dysplasia
Small intestine		Small intestine	
Inhalation		Oral	
Control	0/10 (0%)^Aa^	Control	0/10 (0%)^Aa^
2,4-D LI	0/10 (0%)^Aa^	2,4-D LO	0/10 (0%)^Aa^
2,4-D MI	2/10 (20%)^Ab^	2,4-D MO	8/10 (80%)^Ab^
2,4-D HI	4/10 (40%)^Ac^	2,4-D HO	9/9 (100%)^Ab^
Large intestine		Large intestine	
Inhalation		Oral	
Control	0/10 (0%)^Aa^	Control	0/10 (0%)^Aa^
2,4-D LI	0/10 (0%)^Aa^	2,4-D LO	0/10 (0%)^Aa^
2,4-D MI	0/10 (0%)^Aa^	2,4-D MO	2/10 (20%)^Ab^
2,4-D HI	5/10 (50%)^Ab^	2,4-D HO	8/9 (89%)^Ac^

Data are reported as number and percent. 2,4-D LI: Group exposed to low concentration of 2,4-dichlorophenoxyacetic acid (2,4-D) by inhalation; 2,4-D LO: Group exposed to low concentration of 2,4-D orally; 2,4-D MI: Group exposed to the moderate concentration of 2,4-D by inhalation; 2,4-D MO: Group exposed to the moderate concentration of 2,4-D orally; 2,4-D HI: Group exposed to high concentration of 2,4-D by inhalation; 2,4-D HO: Group exposed to a high concentration of 2,4-D orally. Upper-case letters compare the inhaled and oral groups within the same concentration. Lower-case letters compare different concentrations within the same exposure route. Different letters indicate P<0.05 (Kruskal-Wallis test).

No congestion, inflammation, tissue necrosis, vascular necrosis, atrophy or hyperplasia of the epithelium, lymphoid hyperplasia, or neoplastic lesions were observed in either the small or large intestines in any of the animals evaluated.

## Discussion

In the present study, we observed esophageal hyperkeratosis in most animals exposed to 2,4-D (regardless of route or concentration). Animals exposed to moderate and high concentrations of 2,4-D presented mild intestinal dysplasia, which predominated in those exposed by the oral route. No gastric histopathological changes were observed in any of the animals exposed to 2,4-D.

The threshold limit value (TLV) for 2,4-D is 10 mg/m^3^ for an 8-h time-weighted average exposure. The reference dose (RfD: “an estimate of the quantity of chemical that a person could be exposed to every day for the rest of their life with no appreciable risk of adverse health effects”) for 2,4-D is 0.01 mg·kg^-1^·day^-1^ and the maximum contaminant level (MCL) for 2,4-D in drinking water is 0.07 mg/L (2). According to the European Food Safety Authority (EFSA) (14), 43.4% of food samples from the European Union contained pesticide residues within the permitted range and 1.5% exceed the limits for pesticide concentrations in food. Thus, although inhalation is the most common exposure route to pesticides, the effects of eating food contaminated with pesticides is also important. For this reason, our study examined both inhalation exposure (more common in occupational exposure) and oral exposure (ingestion of contaminated food).

Several toxicological studies suggest that exposure to 2,4-D causes adverse health effects in animals and humans, but how 2,4-D causes toxicity is still unclear. Increased oxidative stress from exposure to 2,4-D is one of the proposed mechanisms for the various changes that this herbicide can cause in the mammalian organism (15).

The epithelia of the oral mucosa and the esophageal mucosa are similar. In humans, both are non-keratinized stratified squamous epithelia (16). In a study with rats chronically exposed to 2,4-D by oral and inhalation routes, hyperkeratosis of the dorsum of the tongue was observed only in animals orally exposed to high concentrations (11). Hyperkeratosis of the tongue, lips, and oral mucosa was also observed in a study that evaluated workers who manufactured herbicides of the chlorophenoxide class, to which 2,4-D belongs (17). In our study, hyperkeratosis was found in the esophageal epithelium of most animals exposed to 2,4-D regardless of the dose and route of exposure. Hyperkeratosis is the thickening of the stratum corneum of the stratified squamous epithelia, characterized by an increase in the keratinization of these epithelia. Hyperkeratosis is an adaptive alteration to chronic irritation of the epithelium by physical or chemical agents (16). Our findings show that 2,4-D attacked the esophageal epithelium, causing an adaptive response, and that the degree of irritation was independent of the concentration and route of exposure, unlike the oral epithelium.

While studies carried out with rodents evaluating the oral cavity after acute inhalation exposure (18) and chronic oral and inhalation exposure (11) showed an increase in tongue epithelium thickness, mainly with an increase in the concentration of 2,4-D, our study did not show an increase in esophagus epithelial thickness. Instead, in our study, 100% of the animals in the groups exposed to the moderate oral concentration and the high inhalation concentration of 2,4-D had hyperkeratosis, and these were the groups with the lowest epithelial thickness. This may have been due to greater damage to the esophageal epithelium, with greater destruction of cells, and even with hyperkeratosis, there was no increase in epithelial thickness. Another fact that corroborates this hypothesis is that the animals exposed to the high concentration of 2,4-D, regardless of route, presented the lowest number of NORs. Our data also contrasted with data from another study, where chronic exposure to high concentrations of 2,4-D was associated with an increase in NORs in the tongue of rats chronically exposed to this herbicide (11). The tissues of the oral cavity are partially protected against chemical agents by the saliva released from salivary glands. Studies show that 2,4-D is transported into saliva, which is a potential candidate for non-invasive human biomonitoring instead of urine (19,20). This may explain the differences observed in our study in relation to the presence of hyperkeratosis, changes in epithelial thickness, and number of NORs. Our data showed that 2,4-D was more toxic to the esophageal epithelium when chronically exposed, independent of exposure route, and stimulated a pattern of adaptive response to irritation different from that observed in the oral epithelium.

In a study of 114,562 Vietnam War veterans using a self-reported disease questionnaire, high exposure to “agent orange” was associated with a significantly higher prevalence of cancer, including colon cancer. In that same study, there was an increased perception of several other diseases, such as circulatory, respiratory, and digestive diseases. Among the digestive diseases, gastritis, peptic ulcer, enterocolitis, gallstones, cirrhosis, and chronic hepatitis were mentioned, but the authors emphasize that these results must be interpreted with caution (21). In another study that is part of the Korean Veterans Health Study, which analyzed the morbidity and mortality of 2,051,098 people who served in the military in Vietnam, it was found that exposure to “agent orange” was associated with a significant increase in stomach and small intestine cancer risk but not esophageal cancer risk. However, these neoplasms were related to exposure to 2,3,7,8-tetrachloro-dibenzo-p-dioxin (TCDD), a known human carcinogen that is a contaminant of 2,4,5-T, which is one of the components of “agent orange” (5). 2,4-D is considered by IARC to be in Group 2B (limited evidence of carcinogenicity to humans; 22). In the present study, we did not observe gastric changes, neither inflammatory nor neoplastic, nor did we observe dysplasia or esophageal neoplasia. This shows that the changes in veterans were probably related to other components of “agent orange” and not to 2,4-D. However, we observed dysplastic lesions in the small and large intestines, which, although mild, showed the carcinogenic potential of 2,4-D for this organ. Although we did not observe a significant difference between the incidence of dysplastic lesions in the small and large intestines at the different concentrations and routes of exposure used, we found dysplastic lesions in the small intestine after exposure to moderate concentrations, which suggested that the small intestine may be more susceptible to 2,4-D than the large intestine.

The effects of 2,4-D on the immune system are controversial, with some studies showing that this herbicide can have immunosuppressive effects and others finding that it has a stimulating effect on lymphocyte proliferation (23). In a study evaluating the intestinal intramural lymphoid tissue of mice that were treated with the immunosuppressant azathioprine and 2,4-D, a similar immunosuppressive effect was observed for the two substances and the effect of azathioprine was potentiated when used simultaneously with 2,4-D (24). In our study, we did not observe inflammation or lymphoid hyperplasia in either the small or large intestine, probably because we used lower concentrations of 2,4-D in our study compared to previous studies, which was not enough to stimulate immunoreactivity.

A previous study with *Rattus norvegicus* exposed by gavage to 2,4-D for 15 days showed an increase in myenteric neurons in the colon, which may lead to an increase in acetylcholine expression that stimulates intestinal smooth muscle and increases the chances of eliminating harmful content in the intestinal lumen (25). This demonstrates that further studies are needed to elucidate the mechanisms of 2,4-D toxicity in the intestine.

Based on our data, we concluded that chronic exposure to 2,4-D, especially at moderate and high concentrations commonly used in crops, regardless of the exposure route, led to hyperkeratosis of the esophagus and mild dysplasia of the small and large intestines.

## References

[1] Mello FA, Fagiani MAB, Silva RCR, Nai GA (2019). Pesticides: impacts to the environment and human health. Colloquium Vitae.

[2] Jervais G, Luukinen B, Buhl K, Stone D 2,4-D Technical Fact Sheet. 2008. National Pesticide Information Center, Oregon State University Extension. http://npic.orst.edu/factsheets/archive/2,4-DTech.html.

[3] (2005). EPA 738-R-05-002.

[4] Dakhakhni TH, Raouf GA, Qusti SY (2016). Evaluation of the toxic effect of the herbicide 2, 4-D on rat hepatocytes: an FT-IR spectroscopic study. Eur Biophys J.

[5] Yi SW, Ohrr H (2014). Agent Orange exposure and cancer incidence in Korean Vietnam veterans: a prospective cohort study. Cancer.

[6] National Academies of Sciences, Engineering, and Medicine; Health and Medicine Division; Board on Population Health and Public Health Practice; Committee to Review the Health Effects in Vietnam Veterans of Exposure to Herbicides (Eleventh Biennial Update) (2018). Veterans and Agent Orange: Update 11 (2018). https://www.ncbi.nlm.nih.gov/pubmed/30629395.

[7] Mello FA, Quinallia G, Marion AL, Jorge FC, Marinelli LM, Salge AKM (2018). Evaluation of the nasal cavity mice submitted to the inhalation exposure to the herbicide 2,4-dichlorophenoxyacetic acid [in Portuguese]. Medicina (Ribeirão Preto).

[8] Bonfim DJP, Magalhães LR, Chagas PHN, Serra FM, Benatti LAT, Nai GA (2020). Hepatic, renal, and pancreatic damage associated with chronic exposure to oral and inhaled 2,4-dichlorophenoxy acetic acid (2,4-d): an environmental exposure model in rats. Comp Clin Pathol.

[9] Nai GA, Filho MAG, Estrella MPS, Teixeira LDS (2015). Study of the influence of the pH of water in the initiation of digestive tract injury in cadmium poisoning in rats. Toxicol Rep.

[10] Rodrigues MAM (2004). Barret's esophagus and dysplasia: diagnostic criteria [in Portuguese]. J Bras Patol Med Lab.

[11] Parizi JLS, Odorizzi GASM, Sato GMRH, Patrão IB, Nai GA (2020). Oral mucosa changes associated with chronic oral and inhalation exposure to 2,4-dichlorophenoxiacetic acid (2,4-D) in Wistar rats. Toxicol Res.

[12] Ploton D, Menager M, Jeannensson P, Himberg G, Pigeon F, Adnet JJ (1986). Improvement in the staining and in the visualization of the argyrophilic proteins of the nucleolar organizer region of the optical level. Histochem J.

[13] Espírito-Santo H, Daniel F (2015). Calculating and reporting effect sizes on scientific papers (1): P<0.05 limitations in the analysis of mean differences of two groups. Port J Behav Soc Res.

[14] European Food Safety Authority (EFSA) (2016). Chemicals in food 2016.

[15] Islam F, Wang J, Farooq MA, Khan MSS, Xu L, Zhu J (2018). Potential impact of the herbicide 2,4-dichlorophenoxyacetic acid on human and ecosystems. Environ Int.

[16] Robbins SL, Cotran RS, Kumar V, Robbins, Cotran (2016). Digestive Tract Pathology. Esophagus. Pathology: pathological bases of diseases.

[17] Chemikosova TS, Kamalova OA, Ibragimova ZN (2004). Status of the buccal mucosa in subjects occupationally exposed to chlorophenoxyherbicides [in Russian]. Stomatologiia (Mosk).

[18] Parizi JLS, Tolardo AJ, Lisboa ACG, Barravieira B, de Azevedo Mello F, Rossi RC, Nai GA (2020). Evaluation of buccal damage associated with acute inhalation exposure to 2,4-dichlorophenoxyacetic acid (2,4-D) in mice. BMC Vet Res.

[19] Carver ZA, Han AA, Timchalk C, Weber TJ, Tyrrell KJ, Sontag RL (2018). Evaluation of non-invasive biomonitoring of 2,4-Dichlorophenoxyacetic acid (2,4-D) in saliva. Toxicology.

[20] Han AA, Timchalk C, Carver ZA, Weber TJ, Tyrrell KJ, Sontag RL (2019). Physiologically based pharmacokinetic modeling of salivary concentrations for noninvasive biomonitoring of 2,4-dichlorophenoxyacetic acid (2,4-D). Toxicol Sci.

[21] Yi SW, Ohrr H, Hong JS, Yi JJ (2013). Agent Orange exposure and prevalence of self-reported diseases in Korean Vietnam veterans. J Prev Med Public Health.

[22] International Agency for Research on Cancer (IARC) (1987). Chlorophenoxy herbicides (Group 2B). IARC Working Group on the Evaluation of Carcinogenic Risks to Humans.

[23] International Agency for Research on Cancer (IARC) (2015). DDT, Lindane, and 2,4-D. IARC Monographs on the Evaluation of Carcinogenic Risks to Humans.

[24] Sapin MR, Lebedeva SN, Zhamsaranova SD, Erofeeva LM (2003). Comparative analysis of disorders in duodenal lymphoid tissue of mice treated with azathioprine and herbicide 2,4-dichlorophenoxyacetic acid and their correction by plant and animal origin remedies [in Russian]. Morfologiia.

[25] Nanni W, Porto GDS, Pereira JNB, Gonçalves ARN, Marinsek GP, Stabille SR (2022). Evaluation of myenteric neurons in the colon of rats exposed to 2,4 dichlorophenoxyacetic acid herbicide. J Environ Sci Health B.

